# Surface Modification of Gutta-Percha for the Use of Intact MTA as a Root Canal Sealer

**DOI:** 10.3390/jfb17060294

**Published:** 2026-06-14

**Authors:** Nastiti Sarilaksmi, Futami Nagano-Takebe, Masatoshi Takahashi, Takashi Kado, Kazuhiko Endo, Takashi Nezu

**Affiliations:** 1 Division of Biomaterials and Bioengineering, School of Dentistry, Health Sciences University of Hokkaido, 1757 Kanazawa, Tobetsu-cho 061-0293, Hokkaido, Japan; nsarilaksmi@lecturer.undip.ac.id (N.S.); nagano23@hoku-iryo-u.ac.jp (F.N.-T.); endo@hoku-iryo-u.ac.jp (K.E.); 2 Department of Dentistry, Faculty of Medicine, Universitas Diponegoro, Tembalang, Semarang 50275, Indonesia; 3 Division of Dental Education Development, Department of Integrated Dental Education, School of Dentistry, Health Sciences University of Hokkaido, 1757 Kanazawa, Tobetsu-cho 061-0293, Hokkaido, Japan; kado@hoku-iryo-u.ac.jp

**Keywords:** mineral trioxide aggregate, gutta-percha, cetylpyridinium chloride, surface modification, contact angle

## Abstract

This study aimed to use intact mineral trioxide aggregate (MTA) as a root canal sealer by hydrophilizing the gutta-percha (GP) surface. The GP specimens were treated with atmospheric air plasma, cetylpyridinium chloride (CPC), or a combination of both. The wettability and surface chemical properties were evaluated using contact angle measurements and X-ray photoelectron spectroscopy (XPS). The physicochemical properties of MTA mixed with water or 100 mM of CPC solution were evaluated using setting time, flowability, compressive strength, and X-ray diffraction (XRD) analyses. Sealing ability was assessed by evaluating the dye penetration in obturated single-rooted teeth. Combined plasma and CPC treatment significantly decreased the contact angle of GP compared to that of the untreated group (*p* < 0.05) and showed the least hydrophobic recovery after 8 weeks. The XPS analysis confirmed the adsorption of CPC onto the GP surface. The XRD and compressive strength results indicated that the CPC did not interfere with the setting reaction of intact MTA, although the setting time was prolonged (*p* < 0.05). Dye penetration was significantly reduced in the plasma- and CPC-treated GP groups compared to the untreated GP group (*p* < 0.05), with a sealing ability comparable to that of the zinc oxide-based sealer.

## 1. Introduction

Root canal obturation is an essential step in endodontic treatment in which the instrumented root canal is filled with an endodontic sealer and core obturation material to prevent microorganism contamination [1,2]. Endodontic treatment has demonstrated a high success rate, ranging from 85 to 95% [2]. However, inadequate cleaning, shaping, and obturation are recognized as important causes of endodontic treatment failure [3,4]. Therefore, achieving an adequate seal throughout the root canal system is critical for preventing reinfection and ensuring long-term treatment success [3,4,5]. Good obturation can be achieved when there is good affinity between the sealer and core obturation material [1,6].

Gutta-percha (GP) is the most commonly used core obturation material for root canal obturation [3,4,7]. The chemical structure of GP is 1,4-trans-polyisoprene, which is the stereoisomer of the natural rubber cis-isomer of polyisoprene [8,9]. However, GP has poor adhesion [2,9,10] and hydrophobicity [11], which may compromise the quality of obturation. To provide a good seal, GP should have a good affinity for the dentinal walls and sealer [6].

Mineral trioxide aggregate (MTA) was first introduced as a root-end filling material for endodontic treatment and has been proposed for pulp capping, apexification, and perforation repair [7,12,13]. Due to its excellent biocompatibility and bioactivity, MTA has been clinically used for root canal filling [1]. However, obturation using MTA alone is not as satisfactory as obturation with GP, because MTA cannot fill anatomical irregularities and apical areas [13]. Therefore, the use of MTA as a sealer instead of as an obturation material has been proposed.

Currently, additional components are added to MTA-based sealers to improve their properties. Methylcellulose and calcium chloride were added to shorten setting time [12], whereas propylene glycol was added to improve handling [14]. Other components have also been reported to influence cell viability [15] and cause severe cytotoxicity in human gingival fibroblasts [16]. Altering the MTA formulation may affect its properties. Hence, it is advisable to use intact MTA as a sealer.

Surface modification has become an option in the medical field to improve biomaterial properties without altering the bulk properties [10,17]. Plasma treatment is an economical and effective surface modification method for improving the wettability and surface roughness [18], leading to improved material adhesion [17]. However, surface wettability regains its original condition within a short time [17]. Furthermore, surfactants can improve the wettability of the materials. A previous study reported that cetylpyridinium chloride (CPC), a cationic surfactant, decreases surface tension and results in improved wettability of the root canal [19]. CPC may enhance the wettability of GP to achieve a better affinity with intact MTA.

This study aimed to use MTA as an endodontic sealer without altering its bulk material by modifying its GP surface. The wettability of the GP and its surface chemical properties were analyzed. Additionally, the physicochemical properties of MTA were evaluated to confirm whether the surface modification method had any influence. Finally, the interfacial sealing performance was evaluated under clinically relevant conditions. The null hypothesis was that plasma and CPC treatment of GP would not affect its wettability, interfacial adaptation, or sealing performance when used with intact MTA as a root canal sealer.

## 2. Materials and Methods

### 2.1. Gutta-Percha (GP) Specimens

Flattened GP specimens (1.5 mm thick) were prepared by pressing a GP bar (Gutta-percha Bar Plus; GC, Tokyo, Japan) between two heated glass plates with a pair of spacers. A flattened-surface GP was used instead of a GP point (GPP), according to previous GP wettability studies [6,7]. The specimens were divided into four groups: control (no surface treatment), atmospheric air plasma treatment (PT), immersion in 100 mM CPC solution (CT), and atmospheric air plasma treatment followed by immersion in 100 mM CPC solution (PC). For each group, nine specimens were prepared for contact angle measurements, whereas four independent specimens were prepared for XPS analysis and four independent specimens for XRD analysis.

### 2.2. Surface Treatment

Atmospheric air plasma treatment was performed using a plasma ion bombarder (PIB-10, Vacuum Device, Ibaraki, Japan) at a discharge current of 6 mA and an irradiation time of 1 min.

A 100 mM CPC immersion solution was prepared by dissolving 358 mg of CPC monohydrate (MW 358; Toko Chemical Industry, Tokyo, Japan) in 10 mL of distilled water. The GP specimens were dipped in the solution for 5 min, and the excess solution was removed from the surface using a mild air blow.

### 2.3. Contact Angle and Surface Free Energy (SFE) Analysis

The flattened-surface GP specimen was placed on the flat surface of a contact angle measuring device (Phoenix Alpha, Surface Electro Optic, Seoul, Republic of Korea). A controlled volume of water droplet (5 µL) was dispensed onto the specimen, and a side-view image of the water droplet was immediately captured (*n* = 9). The measurements were conducted immediately after the surface treatment and at two weeks, four weeks, and eight weeks after surface treatment at ambient temperature and relative humidity (RH). The baseline diameter (2*r*) and height (*h*) of each captured image were measured using ImageJ software (version 1.54g; National Institutes of Health, Bethesda, MD, USA). The contact angle was calculated using the following equation:

$$\theta =2\tan^{-1}\left(\frac{h}{r}\right)$$

Surface free energy (SFE) was calculated based on the contact angle obtained using the following equation:

$$W_{sl}=\gamma_{l}\left(1+\cos\theta \right)$$

where the *W_sl_* is the SFE of the solid, *γ_l_* is the SFE of liquids (water), and the *θ* is the contact angle.

### 2.4. X-Ray Photoelectron Spectroscopy (XPS) Analysis

The chemical properties of the surface were evaluated using X-ray photoelectron spectroscopy (XPS) after treatment for up to eight weeks. The XPS spectra, C 1s, O 1s, Cl 2p, and N 1s were obtained using an XPS (ESCA-850, Shimadzu, Kyoto, Japan) with Mg Kα radiation operated at 7 kV accelerating voltage and 30 mA current under a vacuum of 1 × 10^−6^ Pa.

### 2.5. X-Ray Diffraction (XRD) Analysis

The crystalline structures of the MTA powder (TMR-MTA Mielle, Yamakin, Osaka, Japan) and MTA paste prepared using two different mixing liquids (distilled water or 100 mM CPC solution) were analyzed using X-ray diffraction (XRD) with an X-ray diffractometer (MiniFex 600-C, Rigaku, Tokyo, Japan) operated at 40 kV and 15 mA. Four specimens were prepared for each material condition. The samples were scanned within a range of 2θ = 10–70° at a scan speed of 10°/min. Measurements were performed two hours after mixing on day one and on day seven.

### 2.6. Flowability

A flow test was performed by placing the sealer on a glass plate using a 1 mL syringe, and the tip was cut to facilitate dispensing. The sealer was pressed with another glass plate with a 100 g weight on top, as described in ISO 6876:2012 [20]. After 10 min, the diameter of the sealer was measured and recorded as the flowability. MTA specimens were prepared at a powder-to-liquid ratio of 2:1 (*n* = 6). All the measurements were performed at room temperature under ambient laboratory conditions.

### 2.7. Setting Time

MTA specimens were prepared using two different mixing liquids (distilled water or 100 mM CPC solution) at a powder-to-liquid ratio of 3:1. Setting time was measured using a Vicat needle at 37 °C and 100% RH (*n* = 5). The setting time was recorded as the time elapsed from the start of mixing until no indentation was observed on the surface of the specimen.

### 2.8. Compressive Strength

The MTA specimens with a diameter of 5.3 mm and a height of 8 mm were prepared using a cylindrical mold and two different mixing liquids (distilled water or 100 mM CPC solution) at a powder-to-liquid ratio of 3:1. The specimens were maintained at 37 °C and 100% RH for 2 h to allow initial setting. After initial setting, the specimens were removed from the mold, immersed in distilled water, and stored in sealed glass vials at 37 °C until testing.

Compressive strength was measured using a universal testing machine (Autograph AG-IS, Shimadzu, Kyoto, Japan) at a crosshead speed of 1 mm/min. Measurements were conducted on Days 1 and 7 (n = 5 per group).

### 2.9. Root Canal Specimen Preparation

Experiments using extracted teeth were approved by the Ethics Committee of the Health Sciences University of Hokkaido (Approval No. 260). Written informed consent was obtained before specimen collection.

Single-rooted teeth without caries or signs of fracture were used in this study. The teeth were decoronated and standardized to a length of 15 mm using a low-speed saw (Isomet, Buehler, Lake Bluff, IL, USA). The working length was 1 mm shorter than the root length. To evaluate the interface between GP, sealer, and root canal walls under a clinically relevant root canal filling procedure, root canal obturation was performed using a single-cone technique. The root canals were instrumented with K-files 15–80 (Mani, Tokyo, Japan) using a standard technique. The canals were enlarged to size #80 to standardize the insertion of a #80 GPP without excessive deformation or compaction, rather than to simulate a specific clinical indication. GPP #80 (Zipperer, Munich, Germany) was then used for single-cone obturation. Following root canal preparation, the specimens were pooled and randomly assigned to five groups, as listed in Table 1. Each group consisted of three specimens (*n* = 3). Groups A–D used MTA as a sealer with different surface treatments of the GPP, with Group A serving as the control. Group E used a conventional zinc oxide-based sealer (Canals-N, GC Showa Yakuhin, Tokyo, Japan) as a reference for comparison with a clinically established sealer system. After obturation, the specimens were maintained in an incubator at 37 °C and 100% RH for 24 h to allow the sealer to set.

### 2.10. Evaluation of Interfacial Sealing

To evaluate whether surface modification of GP improves the interfacial adaptation and sealing ability between GP and MTA sealer, dye penetration was assessed from the coronal aspect rather than the apical aspect. A dye reservoir was constructed on the coronal surface of each specimen using wax. In total, 0.25 mL of 0.1% methylene blue solution was placed in the reservoir and maintained at 23 °C and 100% RH for 7 days before dye penetration observation. All the specimens were rinsed with water for 1 min and allowed to dry. The specimens were embedded in an acrylic resin block and ground cross-sectionally using a grinder (Ecomet 3, Buehler, Lake Bluff, IL, USA) at a rotational speed of 130 rpm and a load of 4 pounds, with abrasive paper #600, then finished with #1000. A microscope (MA200, Nikon, Tokyo, Japan) at magnification of 50× and 100× was used to observe the coronal aspect of the specimens at 100 µm intervals. The obtained images were adjusted for contrast and brightness using ImageJ software. The presence of methylene blue penetration, sealer voids, and gaps between the sealer and GP was evaluated. Dye penetration was defined as the distance from the coronal surface to the deepest point of methylene blue penetration.

### 2.11. Statistics

All data were statistically analyzed based on the verification of distribution and homogeneity using SPSS version 26 (IBM, Armonk, NY, USA). For normally distributed data, one-way analysis of variance (ANOVA) was used following Tukey’s post hoc test when the data showed equal variances, or the Games–Howell post hoc test otherwise. For comparison of two groups that were not normally distributed, the Mann–Whitney U test was applied as a non-parametric method. A *p*-value < 0.05 was considered statistically significant.

## 3. Results

### 3.1. Wettability of Gutta-Percha (GP)

**Figure 1:**
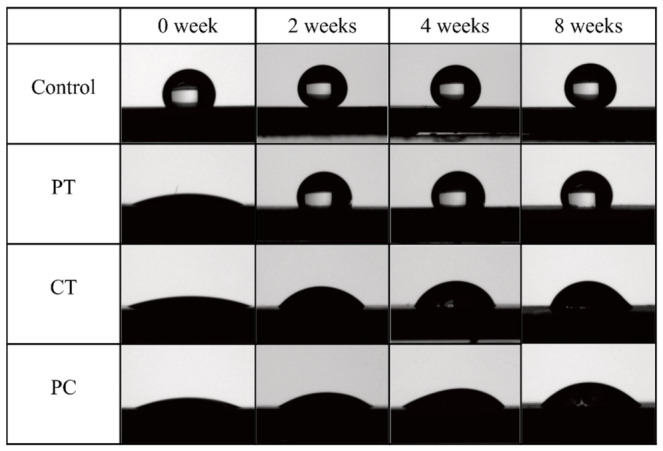
Representative contact angle images of water droplets on gutta-percha surfaces after different surface treatments. PT: atmospheric air plasma treatment; CT: immersion in 100 mM CPC solution; PC: atmospheric air plasma treatment followed by immersion in 100 mM CPC solution.

**Figure 2:**
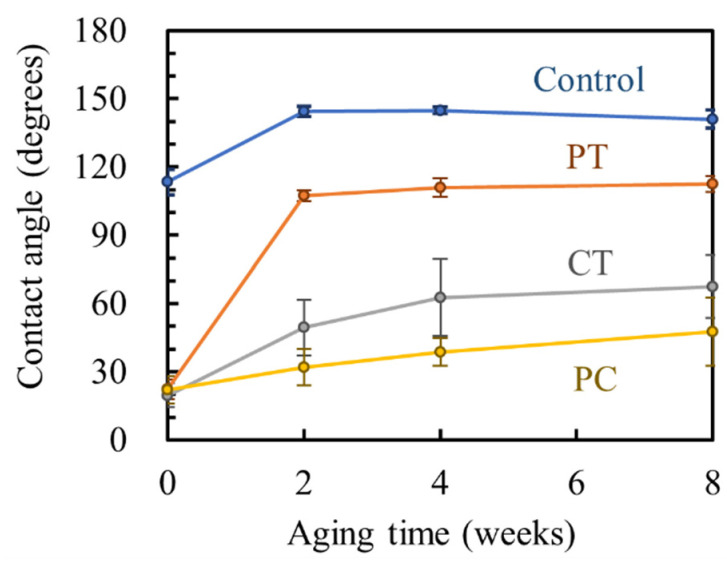
Water contact angle (degrees) of flattened gutta-percha specimens at different time points. PT: atmospheric air plasma treatment; CT: immersion in 100 mM CPC solution; PC: atmospheric air plasma treatment followed by immersion in 100 mM CPC solution.

Representative images obtained during contact angle measurement are shown in Figure 1. Figure 2 shows the contact angles of the control, PT, CT, and PC groups at various time intervals. The contact angles of the PT, CT, and PC groups measured just after the treatment were 22.21° ± 4.42, 19.54° ± 5.24, and 21.99° ± 6.08, respectively, which were significantly lower (*p* < 0.05) than that of the control group (113.27° ± 5.64).

Long-term observations showed that the PT group at week 2 was not significantly different (*p* > 0.05) from the control group immediately after treatment. Meanwhile, the PC group remained significantly lower (*p* < 0.05) than the control group, even after 8 weeks of aging.

Table 2 shows the SFE of the Control, PT, CT, and PC groups during the 8-week aging period. An improvement in the SFE was observed in the surface-treated groups (PT, CT, and PC). Similar to the contact angle results, the SFE decreased over time.

### 3.2. X-Ray Photoelectron Spectroscopy (XPS)

**Figure 3:**
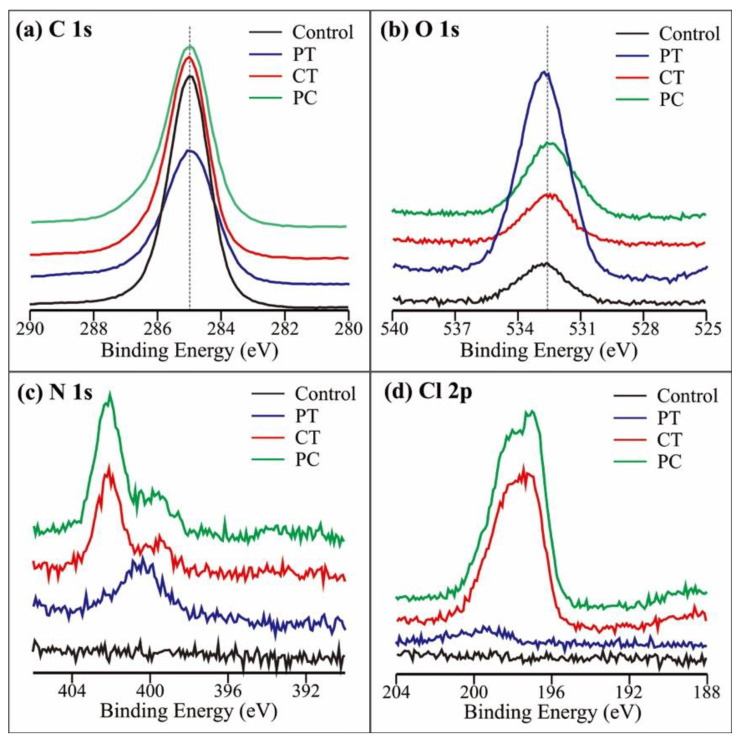
XPS spectra of (**a**) carbon (C 1s), (**b**) oxygen (O 1s), (**c**) nitrogen (N 1s), and (**d**) chlorine (Cl 2p) of flattened gutta-percha specimens immediately after surface treatment. PT: atmospheric air plasma treatment; CT: immersion in 100 mM CPC solution; PC: atmospheric air plasma treatment followed by immersion in 100 mM CPC solution.

**Figure 4:**
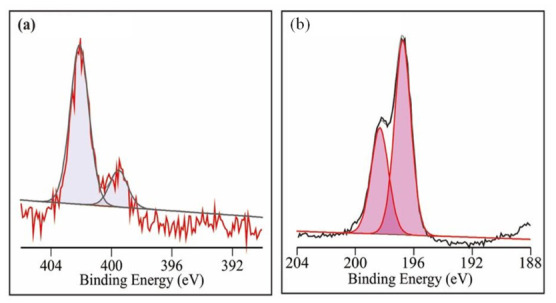
XPS spectra of pure CPC: (**a**) N 1s and (**b**) Cl 2p. Both spectra consist of a pair of larger and smaller bands.

Figure 3 shows the C 1s, O 1s, N 1s, and Cl 2p spectra obtained immediately after the surface treatment. C 1s and O 1s peaks were observed in all groups at 285.0 eV and 532.7 eV, respectively. The N 1s band with a single peak of the PT group was identified at 400.5 eV, which corresponds to -NH_2_. For the CT and PC groups, the N 1s band consists of a main peak at 402.2 eV and a subpeak at 399.5 (CT) or 399.9 (PC). The Cl 2p band has a peak at 197 eV with a shoulder at a higher binding energy. The Cl 2p band is deconvoluted into two pairs of larger and smaller bands at lower and higher binding energies, respectively. Figure 4 shows the N 1s and Cl 2p spectra of the pure CPC. These spectral features resembled those of the CT and PC groups.

### 3.3. CPC Influence on the Physicochemical Properties of MTA

Table 3 shows the physicochemical properties of MTA mixed with water and a 100 mM CPC solution. Among the tests conducted, only the setting time showed a significant difference (*p* < 0.05). A lower compressive strength was observed in the CPC group on day 1, and a higher compressive strength was observed on day 7. However, there was no significant difference (*p* > 0.05) in compressive strength.

**Figure 5:**
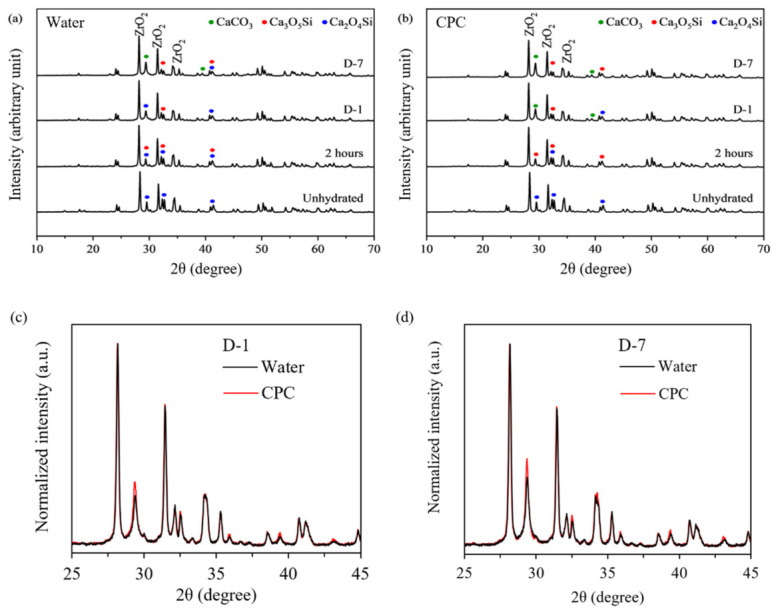
XRD patterns of MTA powder and hydrated MTA mixed using (**a**) water and (**b**) 100 mM CPC solution. Normalized overlay plots of the XRD patterns obtained from MTA mixed using water and 100 mM CPC solution at (**c**) Day 1 and (**d**) Day 7. The traces in (**c**,**d**) were normalized to the ZrO_2_ peak at 28.3°.

The XRD patterns of the powdered MTA and two MTA pastes are shown in Figure 5. The large peaks at 28.3° and 31.4°, obtained from the powdered MTA and both MTA pastes prepared with different solutions, were assigned to ZrO_2_. The smaller peaks at 30.1°, 32.6°, 34.4°, and 41.3° are assigned to Ca_3_O_5_Si and Ca_2_O_4_Si, respectively, which are the main components of MTA. Calcite (CaCO_3_), identified at 30.1° and 39.3°, was observed on day 1 in the CPC group and on day 7 in the water group.

### 3.4. Dye Penetration and Section Observation

The dye penetration results are presented in Table 4. Observation of dye penetration revealed that the interface between the sealer and the GPP showed more leakage than that between the sealer and the root canal. Group A showed a significantly higher dye penetration than the other groups (*p* < 0.05). Among Groups B, C, and D, no significant difference was observed compared to Group E (*p* > 0.05). Furthermore, dye penetration at the interface between the sealer and root canal was not significantly different among the experimental groups (*p* > 0.05).

**Figure 6:**
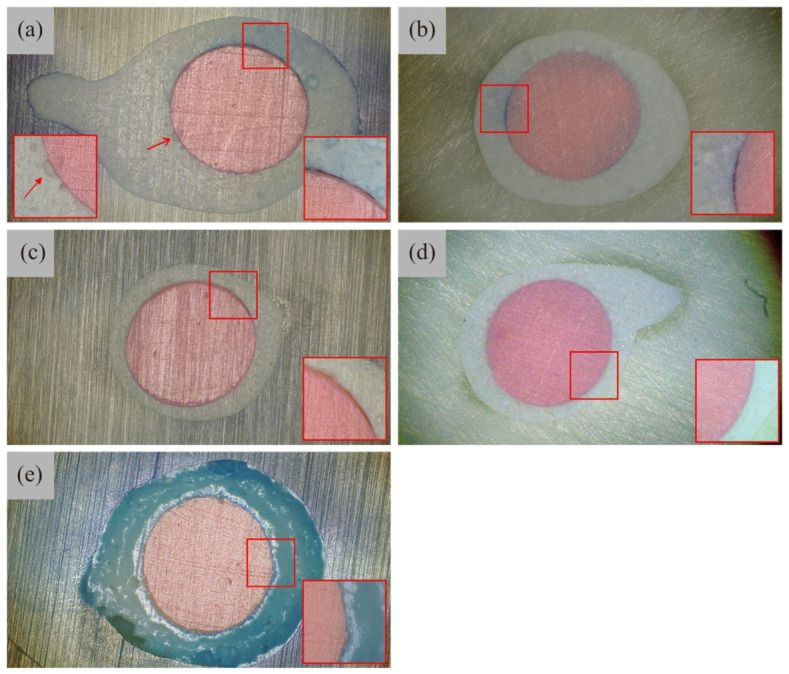
Representative cross-sectional views of specimens at 3 mm from the coronal surface: (**a**) Group A, (**b**) Group B, (**c**) Group C, (**d**) Group D, and (**e**) Group E. The red boxes in the low-magnification images (50×) indicate the regions selected for higher-magnification observation (100×), which are shown as corresponding enlarged images.

Figure 6 shows a cross-sectional view of each group at a depth of 3 mm from the coronal plane. Groups A, B, C, and D had gaps at the interface between the sealer and the GPP. Microscopic observations also revealed void formation at the interface between the sealer and GPP only in Group A. Group E showed good affinity at both interfaces.

## 4. Discussion

### 4.1. Surface Hydrophilization of GP Specimens

The contact angle of the control group was >90°, confirming that the GP was a hydrophobic material. After the surface treatment, the contact angles decreased to <90°, implying that the GP surface became hydrophilic. In this study, the GP surface became hydrophilic after either atmospheric air plasma treatment or immersion in a 100 mM CPC solution. The change in the wettability of GP was also confirmed by the improvement in the SFE of the surface-treated groups. This result is in line with that of a previous study, in which the contact angle of GP decreased, whereas the SFE of GP increased after plasma treatment [10].

The wettability of the PT group decreased immediately after treatment, but recovered to the original condition at week two. The process under which a material surface restores its original hydrophobicity is known as hydrophobic recovery [21]. This result is in agreement with previous studies showing that air plasma treatment results in a rapid increase in the contact angle after aging [17,21]. The polar functional group, NH_2_, introduced on the GP surface, attracts water molecules more easily than hydrocarbons on the GP surface. However, the decrease in -NH_2_ during aging explains the hydrophobic recovery of the PT group.

**Figure 7:**
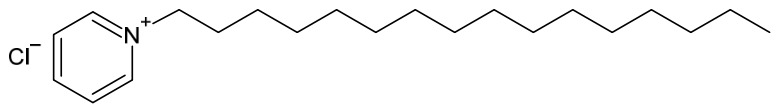
Molecular structure of CPC.

CPC molecule consists of a hydrophilic pyridinium ring with a Cl^−^ counterion and a hydrophobic *n*-C_16_ chain, as shown in Figure 7. Therefore, the N and Cl bands in the XPS spectra of CPC (Figure 4) can be attributed to N^+^ and Cl^−^ in the CPC molecule. Because the N and Cl bands were observed only for the CT and PC groups (Figure 3c,d), the CPC molecules likely covered (or attached to) the GP surface. Therefore, it was concluded that CPC treatment improved the wettability of the CT and PC groups (Figure 2).

**Figure 8:**
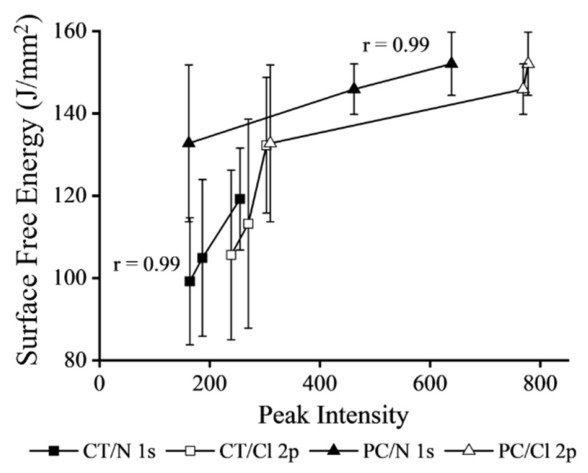
Correlation between surface free energy and the peak intensities of N 1s and Cl 2p. CT: immersion in 100 mM CPC solution; PC: atmospheric air plasma treatment followed by immersion in 100 mM CPC solution.

To clarify the relationship between the wettability and CPC treatment, the SFE (J/m^2^) was plotted against the peak intensity (cps) of the N 1s or Cl 2p spectra for both the CT and PC groups, as shown in Figure 8. There was a strong correlation between the SFE and peak intensity (r = 0.99). This also indicates that the enhanced wettability of the CT and PC groups was caused by CPC attached to the GP surface.

Generally, the increased SFE of the surface-treated groups confirms an improvement in wettability. The SFE is related to the intermolecular bonding at the material surface. Higher energies indicate stronger interatomic attractive forces [9]. CPC is a cationic surfactant with a quaternary ammonium group and chloride counterions in the head group [19]. In this study, a strong correlation was observed between the SFE and the N 1s and Cl 2p peaks of the CT and PC groups (Figure 8). Thus, CPC treatment may have contributed to the increased affinity of GP toward water or a hydrophilic environment due to its surfactant properties. This strongly indicates that the GP surface modified by the treatment methods in this study improves the GP contact with intact MTA, which does not contain organic or polymeric components as modifiers.

In contrast to the PT group, immersion in CPC resulted in less hydrophobic recovery (CT group), and even lower hydrophobic recovery was observed after PT before immersion (PC group). That is, the PC group demonstrated a smaller decrease in wettability during aging than the CT group, indicating that combining both surface modification methods provided a more stable hydrophilic effect.

**Figure 9:**
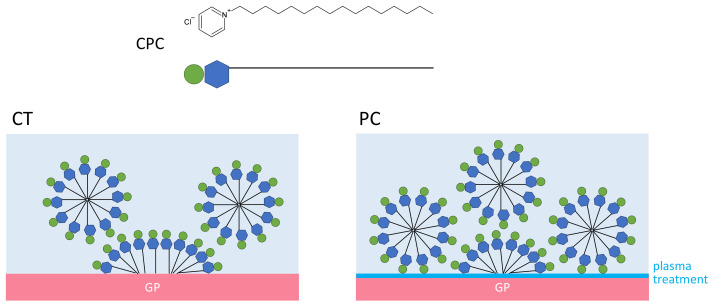
The hydrophilized gutta-percha surface was treated by two different methods. CT: immersion in 100 mM CPC solution; PC: atmospheric air plasma treatment followed by immersion in 100 mM CPC solution.

A possible scheme for the hydrophilization treatment effect based on the present results is shown in Figure 9.

### 4.2. Effect of CPC on the Physicochemical Characteristics of MTA

MTA requires water and a humid environment to initiate the hydration process. Therefore, the presence of CPC on the GP surface may influence this process. The chemical setting reaction of MTA is as follows [1,12,22]:

2(3CaO∙SiO_2_) + 6H_2_O → 3CaO∙2SiO_2_∙3H_2_O + 3Ca(OH)_2_

2(2CaO∙SiO_2_) + 4H_2_O → 3CaO∙2SiO_2_∙3H_2_O + Ca(OH)_2_

**Figure 10:**
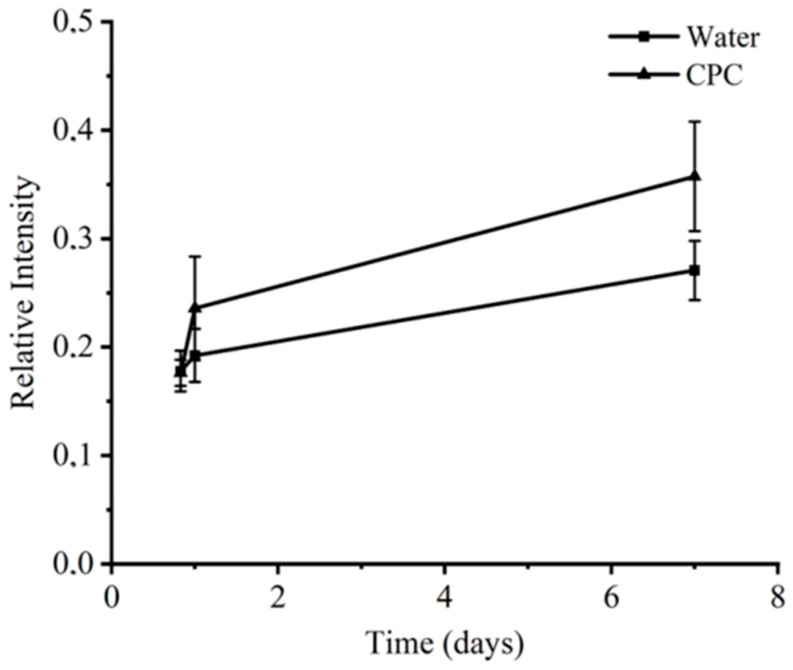
Relative intensity of the indirect MTA setting product, Ca_3_O_7_Si_2_, from the MTA pastes prepared using different mixing liquids (water or 100 mM CPC solution).

The main setting product of MTA, calcium silicate hydrate (C-S-H, Ca_3_O_7_Si_2_∙3H_2_O), was not identified in the XRD pattern because of its nanoscale structure and poor crystallinity, which makes it difficult to detect using XRD [22,23]. Instead, calcite (CaCO_3_) is indirectly monitored during the reaction. Calcite is generated through the reaction of a by-product Ca(OH)_2_ with carbon dioxide in the air during storage [12,23,24]. This result is consistent with previous studies, in which the presence of calcite was reported to have a beneficial effects on the setting process, mechanical properties [24,25], and enhancement of MTA retention by decreasing the porosity and marginal gap [2]. This is consistent with the present compressive-strength test results, in which the CPC group demonstrated greater strength than the water group. Although no significant difference was found in this study (*p* > 0.05), it can be assumed that the higher relative peak intensity of CaCO_3_ (Figure 10) in the CPC group was related to the compressive strength test results.

CPC significantly prolonged the setting time of MTA compared with that of water, as shown in Table 3. This phenomenon was interpreted as follows. During the mixing process, the hydrophilic head of the CPC is adsorbed onto the MTA surface, whereas the hydrophobic tail is oriented toward the opposite aqueous phase [26]. Consequently, water molecules could not approach the hydrophobic MTA surface to initiate hydration. Longer setting times were observed in the CPC group.

The flowability of MTA did not differ between the different mixed liquids. However, neither group complied with the ISO requirements, in which the diameter of the flowability measurement was expected to be >20 mm (Table 3). As an endodontic sealer, it is important to have suitable flowability to seal the gap between the root canal and core materials and fill irregularities in dentin [27]. Moreover, the low flowability of the MTA may have promoted void formation during obturation [1]. Therefore, further studies are required to enhance the flowability of MTA. Nevertheless, CPC did not influence the flowability of intact MTA.

Regarding the compressive strength, CPC had no harmful influence, as the compressive strength was not significantly different between the preparations with the two mixing liquids: water and 100 mM CPC solution (Table 3).

### 4.3. Sealing Ability of the MTA Sealer in Comparison to the ZnO-Based Sealer

Microleakage between the dentinal wall and sealer is usually evaluated using the dye penetration method [28]. However, this technique is unreliable because of limitations in sectioning the specimen on the appropriate side, as the dye does not penetrate symmetrically. Therefore, in this study, dye penetration was evaluated by grinding the specimens at 100 μm intervals and observing them horizontally from the coronal part so that the circumference of the sealer and GP could be evaluated. Moreover, horizontal observation allows the evaluation of the affinity between the sealer and both the GPP and dentinal walls.

Group A showed significantly more leakage at the interface between the MTA and GPP than the other groups. This result is inconsistent with the findings of Ballullaya et al., in which the MTA-based sealer showed the least dye penetration compared to the zinc oxide-eugenol-based sealer [28]. The poor affinity caused by the different properties of the hydrophilic MTA and hydrophobic GP reduces their sealing ability [1]. Furthermore, this condition increased the tendency of the sealer to pull away from the GPP [9,29] and led to void formation, as shown in Figure 6a (red arrow).

The hydrophilicity of GP successfully improved the affinity between MTA and GP, as shown by the smaller gaps observed compared with Group A. The sealing abilities of Groups B, C, and D were consistent with the wettability results, where the surface-treated GP had improved wettability compared to the untreated GP. This result is in line with a previous study in which an improvement in wettability prevented void or gap formation [10]. Furthermore, the sealing abilities of Groups B, C, and D were comparable to those of Group E. Canal-N sealer is a clinically used root canal sealer that is categorized as a zinc oxide-fatty acid-based sealer, which consists of zinc oxide and bismuth subcarbonate in the powder, and fatty acid and propylene glycol in the liquid. Linoleic acid, isostearic acid, and rosin are the fatty acids in Canals-N liquids [7]. Rosin is a natural resin with hydrophobic characteristics [30] that makes Canals-N sealer hydrophobic. The similarity in hydrophobicity between Canals-N and GPP (Group E) provides a good sealing ability. Therefore, the use of intact MTA with surface-treated GP provides favorable clinical results.

Dye penetration at the dentinal wall–sealer interface showed no differences among the groups. Intact MTA has good affinity for dentinal walls owing to its hydrophilicity [1]. Good sealing is provided by the expansion of the material during setting [6,12] and hydroxyapatite formation or mineral infiltration zones on the dentinal walls when in contact with simulated body fluid [1,2,7,28]. The present results demonstrated that plasma and CPC treatment improved the wettability of GP and enhanced its interfacial sealing performance with intact MTA. Therefore, the null hypothesis was rejected.

This study has several limitations. First, interfacial sealing was evaluated using dye penetration and serial cross-sectional observations, and three-dimensional analysis of interfacial gaps and voids using micro-computed tomography (micro-CT) was not performed. Micro-CT may provide additional useful information regarding the spatial distribution of gaps and voids at the sealer–GP interface.

Second, this was an in vitro study and therefore does not fully replicate the long-term clinical environment. Accordingly, further studies incorporating three-dimensional imaging techniques and long-term evaluations would be helpful to further confirm the clinical applicability of the present surface modification method.

In addition, sample sizes for the individual experiments were determined based on previous studies and preliminary experiments, and no formal power analysis was performed. Therefore, the statistical power of the findings should be interpreted with some caution.

## 5. Conclusions

This study’s findings confirmed that the wettability of GP on intact MTA was improved by atmospheric air plasma treatment and immersion in 100 mM CPC without altering the bulk material of GP or MTA composition. Atmospheric air plasma irradiation before immersion in the CPC solution successfully preserved the hydrophilic surface for a longer period by providing more stable adsorption of the surfactant on the GP surface. As a result, hydrophilized GP and intact MTA achieved a favorable sealing ability by preventing void formation and minimizing the interface gap. Furthermore, the CPC surface modification did not interfere with the setting reaction or compressive strength of intact MTA, although a prolonged setting time was observed.

## Figures and Tables

**Table 1:** Sealer and surface treatment used for root canal obturation.

Group	Sealer	Surface Treatment
A	MTA	No surface treatment
B	MTA	PT
C	MTA	CT
D	MTA	PC
E	Canals-N	No surface treatment

PT: atmospheric air plasma treatment; CT: immersion in 100 mM CPC solution; PC: atmospheric air plasma treatment followed by immersion in 100 mM CPC solution.

**Table 2:** Mean ± standard deviation of surface free energy (J/m^2^) of gutta-percha specimens at different time points.

Group	Immediately After Treatment	Week 2	Week 4	Week 8
Control	43.98 (6.52)	13.62 (1.58)	13.33 (1.01)	16.21 (3.33)
PT	139.42 (2.26)	51.23 (2.89)	46.85 (4.85)	44.73 (4.05)
CT	140.55 (2.23)	119.21 (12.37)	104.93 (19.03)	99.23 (15.44)
PC	139.37 (3.12)	134.05 (5.75)	129.43 (4.59)	119.57 (14.28)

PT: atmospheric air plasma treatment; CT: immersion in 100 mM CPC solution; PC: atmospheric air plasma treatment followed by immersion in 100 mM CPC solution.

**Table 3:** Mean ± standard deviation of the physicochemical properties of MTA.

Mixing Liquid	Setting Time (min)	Flowability (mm)	Compressive Strength (MPa)	
			Day 1	Day 7
Water	26.20 (2.17) *	11.90 (0.78)	26.59 (6.67)	35.27 (18.25)
CPC	31.60 (0.89) *	12.31 (1.12)	20.33 (6.90)	38.12 (8.52)

* *p* < 0.05.

**Table 4:** Dye penetration depth (mm) in root canal obturation specimens.

Group	Sealer—GPP	Sealer—Root Canal
A	8.66 (1.29) ^a^	3.41 (2.26) ^A^
B	3.89 (0.22) ^b^	2.06 (0.40) ^A^
C	3.92 (0.62) ^b^	2.43 (0.20) ^A^
D	4.12 (0.48) ^b^	2.82 (0.77) ^A^
E	5.12 (1.41) ^b^	4.57 (0.52) ^A^

Different letters indicate significant differences (*p* < 0.05). Lowercase letters indicate comparisons among groups at the sealer–GPP interface, whereas uppercase letters indicate comparisons among groups at the sealer–root canal interface.

## Data Availability

The original contributions presented in the study are included in the article, further inquiries can be directed to the corresponding authors.
